# Is there a role for proteomics in diabetic renal disease?

**DOI:** 10.1093/ndt/gfz017

**Published:** 2019-02-14

**Authors:** Gemma Currie, Sheon Mary, Christian Delles

**Affiliations:** Institute of Cardiovascular and Medical Sciences, University of Glasgow, Glasgow, UK

## DIABETIC KIDNEY DISEASE

Diabetic kidney disease (DKD) is the most common cause of end-stage renal disease (ESRD). The burden of DKD is expected to continue to increase in parallel with rising prevalence of type 2 diabetes and obesity, coupled with the decline in competing mortality from cardiovascular disease as a result of more effective therapies [1]. Most patients with DKD do not undergo renal biopsy and diagnosis is generally based on clinical parameters, namely presence of diabetes, features of kidney disease including reduced renal excretory function and/or albuminuria, and absence of other kidney disease. However, this pragmatic clinical approach is associated with some uncertainties. Biopsy studies in people with diabetes have shown that approximately two-thirds of patients have superimposed DKD or non-DKD alone and are thereby potentially mismanaged [2]. We know that only a small proportion of patients with diabetes will develop DKD; moreover, the natural history of the disease is evolving. For example, a considerable number of patients develop renal failure without progressing through micro- and macroalbuminuric stages, supporting the concept of non-albuminuric DKD; in addition, patients with microalbuminuria can regress to normoalbuminuria. Histological damage can, however, be present already at microalbuminuric stages.

DKD is a complex and multifactorial condition determined by genetic susceptibility and interaction with environmental factors including glycaemic control, blood pressure and other risk factors such as smoking and use of nephrotoxic medication. Therefore, the prediction of an individual’s likelihood to develop DKD, to progress rapidly to ESRD and to respond to treatment can only be estimated based on features that are thought to play a universal role in the pathogenesis of DKD. It is unlikely that therapies which target all pathogenetic principles simultaneously will ever be available, and personalized approaches to DKD management are therefore warranted. However, we do not currently have the evidence base and the tools to describe individual patients’ disease signature in a manner comparable to rapidly progressing forms of glomerulonephritis, vasculitis or in patients receiving renal transplants where immune diagnostics and molecular pathology inform patient management.

## WHAT IS PROTEOMICS?

Proteomics allows simultaneous quantification of multiple protein markers in a biosample [3, 4]. These markers can be predefined in targeted proteomic approaches that range from antibody-based multiplexing platforms to targeted mass spectrometry such as multiple reaction monitoring. Such platforms assess a limited number of proteins with high precision and often quantitatively. In contrast, untargeted approaches have the potential to assess all proteins in a given sample and are therefore unbiased; in practice, however, methodological constraints limit the number of detectable and identifiable features to several thousands rather than tens of thousands of proteins and modified proteins in biological material. While traditionally gel-based techniques have been used in proteomic studies, there is now an almost exclusive use of mass spectrometry-based methods with higher resolution and faster sample processing [3, 5].

**FIGURE 1:**
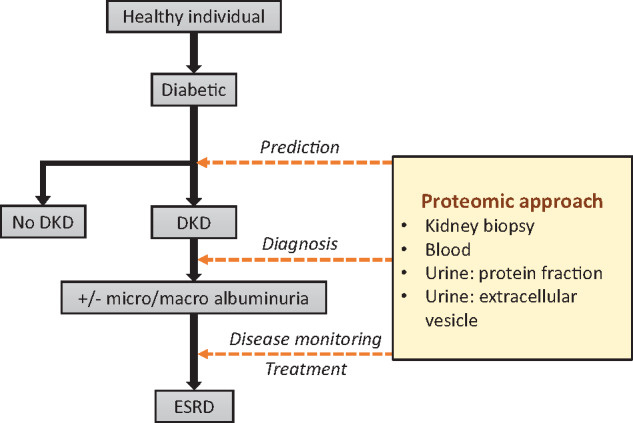
Opportunities for proteomics in the prediction, diagnosis and management of people with DKD. While these opportunities also exist for other biomarkers, proteomic approaches have the potential to cover all aspects of clinical care and offer individual molecular disease characterization and management.

By detecting the proteins that are actually expressed and present in a biological sample, proteomic approaches describe the current state of a cell, tissue or organism better or at least differently compared with genomic and transcriptomic experiments. This is particularly important for dynamic disease processes and monitoring of response to treatment (Figure 1).

## WHICH SAMPLES CAN BE USED FOR PROTEOMICS?

Tissue samples are thought to provide the best possible information on protein expression and the deepest insight into altered physiological processes in a given disease. Newer proteomic techniques such as matrix-assisted laser desorption/ionization-imaging mass spectrometry have the potential to be used for diagnosis [6]. However, in DKD, it is unrealistic to propose that the number of patients who undergo biopsy will significantly increase.

Biofluids including blood can display protein signatures that derive from specific organs including the kidney and also of more generic processes such as fibrosis, which is characteristic not only of advanced renal disease but also of arteriosclerosis or post-myocardial infarction remodelling. Urine is of interest in the study of renal diseases as it is obviously produced by the kidneys. However, protein content is normally low, whereas large amounts of protein appear in the urine unselectively in the event of renal injury as a result of disruption of the glomerular filtration barrier, tubular secretion or reabsorption. Small amounts of specific proteins can be masked by more abundant non-specific proteins such as albumin; this leads to analytical challenges. Moreover, not all urinary proteins derive from the kidney (albumin is a prime example), which makes urine an attractive biofluid for proteomic analyses but reduces its specificity to reflect renal diseases. A major advantage of urine, however, is that it is a rich source of peptides deriving from protease activity upstream and within the kidney that are relatively stable once stored in the bladder. Enzymatic protein digestion is therefore not required in urinary proteomics (more precisely: peptidomics) and samples can be collected, shipped and stored without major precautions [7].

A promising alternative to tissue and biofluids that combine the best of both worlds is the study of extracellular vesicles. These derive from various cells along the nephron and contain cell-specific information that can be transferred into target cells. Their membrane composition and content (cargo) can provide information about their origin, alterations in the molecular (proteomic) make-up of source cells and information that source cells aim to transfer to other cells [8]. Preparation of extracellular vesicles is, however, challenging and has to follow agreed protocols to deliver reliable and reproducible results.

## WHAT ABOUT ANY PREVIOUS AND ONGOING PROTEOMIC STUDIES ON DKD?

Urine is the biofluid of choice for proteomic studies in DKD and has been utilized in studies across the disease spectrum, from early diagnosis to prediction of renal and other outcomes [9–11]. There are, however, a number of limitations of these studies. First, due to the costs related to proteomic studies, sample sizes are often small and data have not always been reproduced in independent cohorts. Secondly, the technology can be complex and sometimes proprietary, which limits widespread clinical adoption of proteomics-based biomarkers. Thirdly, there is no robust gold standard for the definition of DKD, and it is difficult to assess any new biomarker against traditional markers such as albuminuria and renal excretory function with all their limitations.

The most robust data in DKD are available for a classifier composed of 273 urinary peptides detected by capillary electrophoresis coupled to mass spectrometry [12]. The CKD273 classifier was originally developed as a marker of chronic kidney disease (CKD), but has shown promise for early diagnosis and potentially prognosis in DKD. This is plausible since generic pathways involved in other renal diseases such as inflammation and altered extracellular matrix remodelling also play a role in DKD. One could argue that CKD273 represents up to 273 distinct molecular pathways, whereas in reality many of the peptides of which CKD273 is composed derive from collagens and most likely reflect alterations in renal fibrosis. Also, the CKD273 classifier discards thousands of peptides that were not found to be consistently expressed in urine or to be robustly associated with CKD. Some of these may be relevant for individual patients where they indicate patient-specific pathophysiology but will not be reflected by the CKD273 classifier. Large-scale prospective data on CKD273 will soon be available from the Proteomic Prediction and Renin Angiotensin Aldosterone System Inhibition Prevention of Early Diabetic nephRopathy In TYpe 2 Diabetic Patients With Normoalbuminuria (PRIORITY) trial where it has been prospectively employed to predict risk of progression from normoalbuminuria to microalbuminuria [13].

Other urine proteomic approaches are limited to small sample sizes [10, 14] and have not been systematically reproduced. There are also only very limited data from tissue proteomic studies, most of which have been conducted in preclinical models [15, 16].

## IS THERE A ROLE FOR PROTEOMICS IN DIABETIC RENAL DISEASE?

The use of proteomics for early diagnosis of DKD is currently limited by the absence of robust diagnostic criteria. Where precise technology such as mass spectrometry meets imprecise clinical disease definitions, it is practically impossible to improve diagnostic accuracy. The same applies to monitoring disease progression and treatment response, which are currently assessed by albuminuria and estimated glomerular filtration rate. To date, there is no guidance on how treatment would have to be modified in response to changes in proteomic signatures. We believe, however, that post-translational modifications of proteins, namely glycation, may have a role in DKD prediction and monitoring of disease progression. Assessment of variability in glycation pattern is an example of a targeted mass spectrometry approach that could play a role in clinical practice in the future [17]. In contrast, the mantra that omics studies have the potential to unravel new disease pathways and thereby allow development of novel targeted therapies is particularly challenging in complex and multifactorial diseases such as DKD with enormous heterogeneity between patients. There are very few novel biomarkers for diagnosis and management of DKD [18], and none of them has been integrated into widespread clinical practice; the bar is high for novel proteomic biomarkers.

We therefore see the biggest potential for proteomics in phenotyping the molecular basis of an individual’s disease. In other areas of medicine such as oncology, generations of researchers have spent enormous effort on histological and molecular description of different forms of tumours, and the knowledge of molecular heterogeneity of disease has informed application of personalized therapeutic approaches. DKD management lags behind disease management in oncology and in fact other renal diseases, largely because of the lack of human tissue to support molecular medicine. Proteomic studies in urine and other biofluids can help to overcome these limitations in DKD.

While efforts to translate existing proteomic data into clinical practice are laudable, we would in parallel like to see more basic work to develop a disease atlas of DKD describing biomarker profiles and molecular signatures in large numbers of patients. We will very likely see considerable disease heterogeneity, but understanding an individual’s molecular pathophysiology will ultimately allow us to target treatments in the spirit of precision medicine.

## FUNDING

This work was supported by a grant from the European Commission (‘PRIORITY’, grant agreement 101813) and the British Heart Foundation (Centre of Research Excellence Award; reference number RE/13/5/30177). S.M. was supported by a Newton International Fellowship from the Academy of Medical Sciences.

## CONFLICT OF INTEREST STATEMENT

None declared. The results presented in this article have not been published previously in whole or part.
